# Socioeconomic priorities and stunting in rural indonesia: A mixed-method study

**DOI:** 10.1371/journal.pone.0317329

**Published:** 2026-03-03

**Authors:** Akhmad Mustofa, Nanik Suhartatik, Edi Wibowo, Muadz, Ardhea Mustika Sari, Sayit Abdulloh Al Farizi, Lintang Octaviara Pradana, Claradita Mutiara Vannessa, Ananda Mahligai Sukma

**Affiliations:** 1 Department of Food Technology, Faculty of Technology and Food Industry, Universitas Slamet Riyadi, Surakarta, Central Java, Indonesia; 2 Department of Management, Faculty of Economy and Business, Universitas Slamet Riyadi, Surakarta, Central Java, Indonesia; 3 Department of Communication Science, Faculty of Social and Political Science, Universitas Slamet Riyadi, Surakarta, Central Java, Indonesia; 4 Department of Food Science and Technology, Faculty of Agriculture, Universitas Sebelas Maret, Surakarta, Central Java, Indonesia; Kandahar University Faculty of Medicine, AFGHANISTAN

## Abstract

As part of the plan to lower the stunting rate by up to 14% in Indonesia in 2024, a nutritional intervention program for stunted children was implemented in 2023. The program, which ran for four months, involved the daily administration of high-protein supplementary food to 4.9 million stunted children. However, it failed to reduce their number in some districts, including Sukodono, resulting in a high stunting rate of 20.89% (357 toddlers). The aim of this research is therefore to evaluate the major factors related to Supplementary Feeding Program (SFP) inefficiencies in Sukodono by examining the socioeconomic status and lifestyle of the community, and their knowledge of healthy food. A descriptive-exploratory approach with the simple random sampling method was employed. Data were collected through structured interviews with caregivers, observations of community lifestyles, and assessments of caregiver knowledge of healthy food. As demonstrated in the results, out of the 189 participants, 7.4% earned less than IDR 1,000,000.00, implying the necessity for frugal living, especially in the spending of household income. They possessed an adequate understanding of the importance of nutritious food for child growth. 51% were male, and 24% of the parents of stunted children used no form of contraception. Additionally, the parents were relatively young: 50% of the fathers and 46% of the mothers were aged 31–40, while 46% and 49% of the fathers and the mothers respectively were senior high school graduates. Few had continued to tertiary levels, with only 7% of the mothers holding a bachelor’s degree. Efforts by the government are considered necessary to alter community behavior and lifestyles and to highlight the urgency of formal education, as caregivers’ education levels affect children’s health. The study recommendations made include educating caregivers about the significance of fulfilling children’s nutritional needs, promoting healthy feeding practices for toddlers, and better planning of the birthing process.

## Introduction

Indonesia is predicted to experience an increase in the prevalence of stunting, wasting and underweight children based on its demographic conditions and related health surveys [1]. Nevertheless, data from The Indonesian Health Survey 2023 in Number [2] indicate that the prevalence of stunting in the country decreased from 24.4% in 2021 to 21.6% in 2022. Such data encouraged the government of Indonesian to target a reduced stunting rate of 14% by 2024, considering the significant issues stunting poses in the long term, such as premature mortality in children, lower emotional intelligence, and hindrances to cognitive and non-cognitive development in adulthood [3]. Government initiatives included a reduction in child stunting by providing supplementary feeding and nutritional interventions, involving the provision of additional food to children identified as suffering from stunting, wasting or being underweight. The program was simultaneously across all regions in Indonesia for four months.

It should be noted that considering the broad factors contributing to stunting, implementing a stunting intervention program should be tailored to addressing the specific underlying causes. Ignoring this notion would result in the failure of the program. One region that makes a significant contribution to the stunting rate in Indonesia is Sukodono, a sub-district located in Sragen Regency, Central Java. Based on an evaluation conducted by the research team, nutritional intervention in Sukodono has not been effective in addressing stunting [4].

Stunting refers to a condition in which toddlers have a lower height relative to the normal height of children of the same age. Influencing factors include a lack of maternal knowledge about nutrition and health; limited access to healthcare services; inadequate nutritional intake; poor economic conditions; and constrained access to sanitation and clean water. The primary cause of stunting, or inadequate child growth, is the insufficient intake of nutrients, particularly animal protein. The government’s Supplementary Feeding Program (SFP) was designed to ensure balanced nutrition, achieved by providing additional food to stunted children across Indonesia on a daily basis for four months. Food preparation is organized by health cadres from each region. In dealing with 4.9 million children with stunting in Indonesia in 2023, the government needed to deploy substantial funds on mandatory logistics, distribution, monitoring, evaluation, coordination, and reporting. Accordingly, because SFP was costly, it should be reassessed, particularly when it fails to demonstrate effectiveness in lowering the stunting rate.

Yani et al. [5] indicate that direct and indirect factors influence the nutritional status of children. The direct factors are children’s diet, overall health (including whether they have infectious diseases), and physiological conditions, while indirect ones include exclusive breastfeeding, access to healthcare facilities and potable water, family dynamics, lifestyle, parenting practices, and living environment. Another important factor is the educational level of parents or caregivers.

A study conducted by Yazew [6] identified that the causes of stunting in the Jima Geneti District of Ethiopia were low levels of well-being and diets that did not meet nutritional needs. Yazew [6] suggests that interventions should prioritize enhancing mothers’ knowledge and education through effective communication; monitoring the health of pregnant or breastfeeding mothers; and encouraging families to cultivate fruit and vegetables and to raise livestock for protein sources. However, findings from a study by Gholampour et al. [7] present different results, indicating that the causes of abnormal growth in Qazvin, Iran, were low socioeconomic status and insufficient food availability in terms of quality and quantity, affecting family-level food security.

Gholampour et al. [7] propose improving community welfare, generating job opportunities for families, and offering nutritional education to mothers to ensure that families have access to healthy and adequate food. In other words, these two studies argue that interventions to address stunting should be tailored to cope with the specific issues faced by individual regions. Reflecting on the issues occurring in the two countries, it can be argued that Indonesia is experiencing a similar situation; that is, inadequate access to nutritious food, low nutritional education, low availability of clean and decent water, poor parenting standards, poor dietary patterns for children, poverty, limited knowledge of the community concerning stunting, and funding constraints. However, it must be emphasized that the causes of stunting differ between regions, calling for different solutions and treatments.

In Indonesia, a developing country, problem-solving should be grounded on existing problems, which must be clearly understood prior to formulating effective solutions. Related to SFP inefficiencies in Sukodono, the problem may not lie primarily in unavailable access to nutritious food or socio-economic factors, but to another cause. Establishing the fundamental causes of stunting in the district and subsequently implementing effective solutions are crucial. This study, therefore aims to identify the factors contributing to stunting in Sukodono and to offer recommendations to local and central government authorities, enabling more efficient and effective stunting reduction, particularly in Sukodono.

Caregivers’ educational levels are a critical factor in stunting reduction [8–10]. Most children in Indonesia are reared by their parents or other family members, for example, grandmothers or aunts. Mothers who live in big cities and work, earning upper-middle incomes, are likely to hire professional caregivers. Sragen is a rural area mostly populated by farmers and factory workers with low incomes, who accordingly prefer their children to be taken care of by family members, which is considered to be cost-saving. Food consumed by such children is not specifically prepared to suit their needs, but complies with what is eaten by the adults in the house, hence sometimes ignoring children’s specific nutritional needs. This phenomenon occurs due to a lack of knowledge of the caregivers and family of children’s nutrition, showing their lack of attention to the importance of providing healthy food and providing adequate nutrition.

During harvesting, farmers earnings will increase, but they continue to spend little on dietary pattern improvements, particularly for their children. They will use the money for other purposes, such as purchasing motorcycles, repairing their houses, or saving, demonstrating their low awareness of the significance of sufficient nutritional intake for their children. Unfortunately, data on child caregivers’ knowledge levels in Indonesia are scarce. Most of the research on caregivers focuses on those for elderly people or patients with specific diseases [11–14], while research on child caregivers mainly addresses the association between service satisfaction levels and the quality of service of babysitters [15]. This study, therefore seeks to answer the following research question: What are the key socioeconomic, behavioral and knowledge-related factors contributing to the inefficiency of the Supplementary Feeding Program (SFP) in reducing stunting in Sukodono? We hypothesize that caregiver education, lifestyle practices, and maternal health indicators significantly influence stunting outcomes in the region.

## Materials and methods

### Sampling methods

This descriptive-exploratory study was conducted in nine villages in Sukodono, Sragen. The district covers an area of 47.48 km^2^ and is populated by 34,098 people, with a population density of 718.16 people per km^2^ [16]. The Community Health Center (CHC) of Sukodono reported that 357 toddlers were experiencing stunting in July 2024. Family data collected from the CHC included names, addresses and identity numbers. Using Slovin’s formula [17], a sample size of 189 families was determined, as follows:


$$n=\;\frac{N}{1+\;{Ne}^{2}}$$ (1)

where:

n  = the total sample

N  = the population sample (the number of stunted children)

e  = the significance level (0.05)

Consequently:


$$n=\;\frac{357}{1+\;{357x0.05}^{2}}$$ (2)

$$n=\;\frac{357}{1.8925}$$ (3)

    =188.64 ≃ 189 respondents (4)

Using Slovin’s formula with a 5% margin of error, the minimum sample size was calculated as 189 families. Each family was assigned a unique identification number, and a simple random sampling technique was applied using a computerized random number generator to select participants. The process was conducted in collaboration with CHC staff to ensure transparency and minimize selection bias. The family domicile distribution was across the nine villages. Respondents were recruited by health cadres and given explanations concerning the data collection process. They gave their consent verbally, and their willingness to participate was recorded by field workers by marking a check (✓) on the forms provided before proceeding to the next process. Written consent was not obtained due to cultural and logistical constraints in the study area; verbal consent was considered more appropriate and was documented by field staff using standardized forms. The procedure was reviewed and approved by the Ethics Committee of Dr. Moewardi General Hospital (number 2.316/IX/HREC/2024). There was no direct engagement of children in the study, although the questions posed also inquired about their health conditions. The data recorded referred to children’s growth and immunization records, all documented in the mother and child growth logbook; the respondents gave their consent that the research teams could access these. Children suffering from chronic diseases (including HIV/AIDS, congenital heart defects, renal failure or diabetes) based on the medical records at the CHC and caregiver confirmation were excluded from the study.

The research phases included developing a questionnaire through a focus group discussion with 14 stakeholders, comprising 4 public health experts (lecturers and practitioners), 3 national government officials, 1 NGO representative, and 4 health cadres. The FGD lasted approximately 4 hours and was moderated by the research team. Consensus on questionnaire content was reached through iterative discussion; items were proposed, debated, and revised. The questionnaire was pilot-tested and evaluated for internal consistency. The overall reliability coefficient, measured using Cronbach’s alpha, was 0.723, indicating high internal consistency.

### Data collection

#### Socioeconomic study.

Data were collected through family visits, observations, interviews, and questionnaire distribution (N = 189 respondents) from October 5^th^ to 30^th^ 2024. To avoid clustering effects, only one stunted child per household was included in the studies. Observations were made in the environments of respondent’s residence. The data collectors completed forms concerned with data on the building area, the number of residents in the building or the house, the flooring material, main building construction materials (particularly whether the building was well painted and had either plaster, wooden, bamboo or mixed material-based walls), lighting, sources of electricity, private vehicle ownership, fuels used in cooking, and water sources for daily use, amongst others.

Furthermore, data on the respondent’s socioeconomic conditions were collected by referring to 14 criteria established by Statistics Indonesia (BPS) in 2020. If nine of the following 14 categories were met, the household classified as poor. The 14 criteria from BPS were:

1. The household lives in a house with a floor area of less than 8 m^2^ per person.
2. The household lives in a house with a floor made of bamboo, low-quality wood, or without flooring.
3. The household lives in a house with walls constructed of bamboo, thatch, low-quality wood, or non-plastered walls.
4. The household has no access to latrine facilities and shares sanitation with other households.
5. The household does not have electricity for lighting.
6. The household derives its drinking water from unprotected wells, springs, rivers or rainwater.
7. The household uses firewood/charcoal/kerosene as cooking fuel.
8. The household only consumes meat/dairy/chicken once a week.
9. The household head’s highest education is no formal education at all, or graduating or not from elementary school.
10. The household head’s employment status indicates informal or low-paid occupations.
11. The household has inadequate access to healthcare services.
12. The household buys only one new outfit a year.
13. The household eats once/twice day.
14. The household lacks savings or easily sellable items worth at least IDR500,000.00.

#### Demographic study.

In addition to socioeconomic data, the questionnaire investigated the respondents’ demographic information (including the age, weight and height of the toddlers), the number of children in the family, the total number of family members, occupation of the fathers and/or mothers, and family income. It also asked the respondents or caregivers several questions regarding the food given to toddlers to evaluate their knowledge of healthy food.

#### Data collection through interviews.

Data were gathered through interviews with all 189 parents/caregivers, specifically inquiring about how they ensured the availability and adequacy of food for their children. Data collectors conducted face-to-face interviews with household members, including parents, caregivers, grandparents, or siblings. During the interviews, caregivers were asked about access to clean water, healthy lifestyle habits, the availability of latrines, dietary habits, and other pertinent factors. The interviews were conducted using a structured method, preventing data collectors from further developing the list of questions that had been set. Caregiver knowledge and lifestyle were assessed using structured questionnaires. Scores were categorized as “good” when respondents answered ≥80% of items correctly, “adequate” when 60–79% were correct, and “poor” when <60% were correct. In cases where no formal threshold was applied, interpretation was based on the distribution of correct responses across items.

The triangulation method was also applied, and the response validity was confirmed through field observations and data cross-checking using medical history records and children’s health monitoring books. Additionally, the interviews were conducted employing a good communication process, in which the data collectors made an appointment with the respondents, thus allowing for a conducive environment. The interview results served as the basis for our recommendations to the government and policymakers in designing effective interventions for combating stunting. This study employed a convergent mixed-methods design, in which quantitative survey data and qualitative data (structured interviews, field observations, and triangulation with medical records and growth books) were collected in parallel and integrated during interpretation.

#### Data analysis.

Data analysis was performed with a descriptive test using SPSS version 25 software for Windows. Results are presented as percentages, means, standard deviations, and chi-square test statistics with degrees of freedom and p-values. Data analysis was performed using descriptive statistics and chi-square tests to examine associations between categorical variables. Conclusion drawing was undertaken in a deductive manner with reference to the analyzed factors, covering economic, social, and knowledge areas.

## Results and discussion

This study set out to examine whether caregiver education, lifestyle practices, and maternal health indicators influenced stunting outcomes in Sukodono. The analyses revealed that household income and maternal age at childbirth showed significant associations with stunting-related outcomes, while caregiver education level did not demonstrate a statistically significant relationship with knowledge or feeding practices. An important tension in our findings is that caregivers demonstrated relatively high knowledge scores and reported adequate lifestyle practices on structured measures, yet stunting prevalence in Sukodono remains high. This discrepancy suggests that knowledge alone is insufficient to ensure healthy child growth. Socioeconomic constraints, cultural feeding norms, and household spending priorities often limit the translation of knowledge into practice. For example, despite awareness of exclusive breastfeeding recommendations, some caregivers introduced complementary foods early or allowed children to consume junk food. These findings highlight the need for interventions that not only improve knowledge but also address behavioral, cultural, and economic barriers to applying that knowledge in everyday caregiving.

1. Household-level determinants

The statistical description of stunted patients in Sukodono and a comparison with the other districts are shown in S1 Fig, while the comparison between Sragen and other regencies is indicated in S2 Fig. They are annual data, with a cutoff point of December 2024. In S1 Fig, there was a high percentage (6.97%) of stunted children in Sukodono, higher than in other districts apart from Mondokan and Kalijambe. Among the regencies in Central Java, Sragen is ranked seventh, with a stunting rate of 24.3%. Such high rates should be of concern to the government, which should immediately plan effective programs to reduce them. Syafrawati et al. [18] report that the government of Indonesia runs stunting reduction programs by assigning its ministers from the Ministry of Health, the Ministry of Agriculture and Food Security, the Ministry of Marine Affairs and Fisheries, the Ministry of Public Works and Housing, the Ministry of Family Planning, Social Affairs, and Village Community Empowerment, and the Ministry of Regional Development and Planning. However, despite their overarching nature, the programs have a minimal effect in reducing stunting in Indonesia.

The results of stunting reduction in West Sumatra are similar, with several regions standing out for their reduced rates, while others show stagnant outcomes. Factors hampering stunting reduction include a lack of human resources, limited funding for relevant programs, inadequate supporting systems (equipment, standard operating procedures, academic recharge for cadres, and others), and poor communication, specifically at the government level [18]. Beal et al. [19] propose that factors causing stunting in Indonesia include household and family issues, inadequate complementary feeding, unsupportive breastfeeding behaviors, and infection, besides environmental and social factors. It is important to note that Sukodono is not an outlier compared to national stunting patterns; rather, its prevalence aligns with broader trends observed across Indonesia. This reinforces the argument that local contextual factors—such as household socioeconomic conditions, caregiving practices, and maternal health behaviors—play a decisive role in shaping outcomes, even when national supplementary feeding programs are in place.

Observational data on housing conditions and hygiene practices help explain why families with seemingly adequate socioeconomic status continue to experience high stunting rates. Based on data collected in October 2024, 50.79% of the respondents were male (S3a Fig). Most families choose to plan their children’s births through family planning programs, with injections, IUDs, or implants as the preferred methods. Conversely, 23.81% of the respondents opted not to participate in any family planning programs (S3b Fig). Among the 189 households surveyed, 23.81% reported not using any form of contraception. Parents were aged in the range of 31–40 years (S3c and S3d Fig). The age of the fathers was mostly between 31 and 40 years old (50.26%). Similarly, most of the mothers (46.00%) were aged between 31 and 40 years old, with 45% aged 20–30. Generally, the educational level of the mothers was higher than that of the fathers (S3e and S3f Fig). The proportion of mothers with a bachelor’s degree was 6.88%, while only 2.65% of the fathers had reached the same educational level. The fathers’ occupations varied significantly, but the respondents’ three main jobs were laborers (26.46%), entrepreneurs (20.11%), and farmers (17.99%). On the other hand, the primary occupation of the mothers was housewife (70.90%), followed by employee (6.35%) and entrepreneur (5.29%) (S3g Fig).

Considering the characteristics of the parents and comparing them with the data shown in S3 Fig, it can be interpreted that the parents were categorized as being relatively young (under 40). The majority had graduated from junior or senior high schools, but few pursued a bachelor’s degree (3% of the fathers and 7% of the mothers). The educational levels of the parents with stunted children in Sragen can therefore be said to be relatively low. However, based on the Chi-square analysis, caregivers’ education levels had an insignificant impact on their knowledge of healthy food (χ² = 0.354, df = 1, p = 0.552). Laksono et al. [20] demonstrate that mothers’ education levels influence their breastfeeding habits; it is well-known that low breastfeeding habits contribute to a high stunting rate [19].

64.55% of mothers gave birth at the age of 20–30 years, while the figure for those giving birth aged 31–40 was 29.10% (S4 Fig). Most infants were born with a normal birth weight (88.36%), while the remainder had an abnormal one. Children with low birth weight are more likely to be unhealthy or experience growth impairments due to inadequate nutritional intake during the first 1,000 days of their lives [3]. Sartika et al. [3] remark that children born with a low birth weight have a higher tendency to suffer from stunting.

Furthermore, children born with an adequate birth weight, premature birth, mothers with short stature, and incomplete immunizations can also contribute to an increased stunting tendency. Premature birth can be one of the causes of stunting in children. The data indicate that 23 mothers (12.17%) of children suffering from stunting gave birth prematurely. Such a birth refers to a condition when a child has not had sufficient time to develop normally in the womb. The gestational period is a crucial time, since it determines fetal development, so the fetus should therefore receive adequate nutrition during this period. As fetal development follows a specific timeline, prematurely born babies are those who are unable to reach the appropriate stage or time of delivery. Maternal health during pregnancy is hence essential for delivering a healthy child. Meeting nutritional needs during the first 1,000 days is also critical for supporting fetal development, child growth, breastfeeding, and providing sufficient energy for the child and its mother [21].

A significant majority (96.30%) attended health facilities regularly for prenatal check-ups (S4 Fig). The figure for pregnant women consuming iron supplement tablets was 90.48%, while that for folic acid consumption was 91.53%. Folic acid requirements during pregnancy are 5–10 times higher than those of the general female population. Thus, folic acid supplementation is essential during pregnancy as it cannot be synthesized by humans or animals.

Research also indicates that supplementation with folic acid and iron can enhance the health of both the mother and the fetus. The administration of folic acid and iron supplements has been shown to prevent low birth weight, which is a contributing factor to the increased prevalence of stunting [22,23]. Data collected in Sukodono reveal that pregnant women sought prenatal care at nearby health facilities and received iron and folic acid supplementation during their pregnancies as part of a routine government program. Although the quantitative data showed high levels of prenatal care attendance and supplementation, the qualitative interviews revealed gaps in understanding the importance of folic acid and iron, suggesting that access alone may not ensure effective utilization. Meanwhile, 8.47% of pregnant women did not take folic acid. Wiradnyani et al. [24] state that the reason why pregnant women do not take this is their lack of knowledge about its importance in supporting children’s and their own health.

To achieve optimal child growth, it is recommended that infants be exclusively breastfed for the first six months (exclusive breastfeeding). Among the 189 respondents, 4.76% reported introducing complementary feeding to their children before the age of six months, despite the guideline that infants should receive only breast milk up to the age of six months. Exclusive breastfeeding is considered an effective solution for reducing the prevalence of stunting in low- and middle-income countries (LMICs), such as Indonesia [25]. Nevertheless, 41 parents (21.69%) provided complementary feeding to their children when they were over six months. To ensure adequate nutritional intake, additional formula milk was sometimes given, particularly when breast milk production did not meet the child’s needs. Based on the interviews, it was also found that respondents actively visited health facilities to receive vitamin A supplementation (87.83%) and obtain complete immunizations (93.12%). While structured knowledge scores were generally high, our findings suggest that knowledge does not always translate into practice. For example, adequate awareness of healthy feeding, some caregivers reported introducing complementary foods before six months, or allowing children to consume other foods. This highlights the gap between knowledge and behavior, underscoring the need for interventions that address both awareness and practical application. Caregiver education, lifestyle practices, and maternal health indicators are interconnected determinants of stunting.

One of the factors that increases the risk of stunting is the socioeconomic situation. Table 1 presents data on the related status of families with stunted children in Sukodono. Among the 14 criteria for poverty defined by Statistics Indonesia (BPS), four were noticeable in Sukodono: dietary patterns, lifestyle, education, and the quality of residential building construction that did not meet standards (for example, ground/dirt floors). According to the survey, only 70.90% of families were aware that nutritious food should be prioritized. In addition to food, another aspect often overlooked was clothing purchases. The impact of this on the risk of stunting is not significant, but suitable clothes are needed to support hygiene practices.

**Table 1:** Socioeconomic Conditions of Families with Stunted Children.

Socioeconomic Criterion	% Respondents
The floor area of the residential buildings is < 8 m^2^ per person.	0.53
The residential building floor is made of soil/bamboo/cheap wood.	12.70
The residential building walls are made of bamboo/palm leaves/low-quality wood/non-plastered bricks.	15.34
The household has no toilet facilities/shares with other households.	0.00
The household does not use electricity as a lighting source.	0.00
The drinking water sources come from unprotected wells/springs/rivers/rainwater.	22.75
The fuel for daily cooking is firewood/charcoal/kerosene.	3.70
The household only consumes meat/milk/chicken once a week.	29.10
The household only buys one new outfit a year.	28.04
The household can only pay for medical treatment at health centers/clinics.	0.00
In terms of income, the household head works as a farmer cultivating an area of 500 m^2^, is a farm laborer, a fisherman, a construction worker, a plantation worker, and/or does other jobs with an income below IDR600,000.00 per month.	7.41
In terms of the highest educational level, the household head has no formal education/did not finish elementary education/graduated from elementary school.	12.17
The household has no savings/items that can be quickly sold at a minimum value of IDR 500,000.00, such as credit/non-credit motorcycles, gold, livestock, motorboats or other capital goods.	1.06
The household can only eat once/twice a day.	14.29

According to Kuchenbecker et al. [26], the educational level of caregivers influences children’s nutritional status, making it essential to choose those who possess adequate knowledge and education for child-rearing. Most toddlers in Sukodono were cared for by their parents, while some were looked after by their grandmothers or other close relatives. Novianti et al. [27] also reported that the educational level of parents or caregivers affected the prevalence of stunting within families.

The demographic data indicate that awareness of the importance of education within the community was low (S3e and S3f Fig). It is hence essential to provide training to caregivers on appropriate caregiving behaviors and nutritional practices for pregnant and breastfeeding women or children under five years. Setiawan et al. [28] suggest that improvements in dietary habits, lifestyle choices, and cognitive frameworks should commence during adolescence, engendering a clear understanding of healthy behaviors and practices to ensure children’s health from an early age. Besides, because children tend to adopt similar dietary patterns and habits to their parents or those around them, education should also be directed toward parents (families) [29].

Child-rearing among the Sukodono community was affected by other factors, including superstitions and taboos related to child-rearing practices, which could hinder or adversely impact child growth. For instance, the community believes that a mother should avoid fish-based foods for 40 days after giving birth, and that a fussy child should be given complementary feeding, even if the age is not appropriate. Misconceptions about breastfeeding practices, child feeding, and post-partum habits were also found in LMICs [30,31].

Additionally, according to a prevalent perception, a healthy child should be fat, with even overweight children being deemed desirable. Besides, an ideal adult body should be slender, putting pressure on women that are breastfeeding or have recently given birth to maintain a slim figure. Given such thinking, it is crucial to educate adolescents and prospective parents about healthier perspectives on life, child-rearing practices, dietary habits, and the significance of education for improving overall well-being. Mothers should be encouraged to consider their children’s health as the utmost priority and disregard the public stereotypes of the ideal female body, hence increasing their self-confidence regarding their appearance.

The research also suggests that the composition of house floors impacts the prevalence of stunting within families. Approximately 12.70% of families resided in houses with earthen floors. Such flooring provides inadequate support for standard personal hygiene practices, requiring those faced with it to enhance their personal hygiene to mitigate the risk of illness. Such mitigating measures are critical, considering that the presence of an ill family member can negatively influence the health of other household members, especially when the illness is contagious. Children living in unsanitary environments are also more susceptible to illness compared with those in cleaner surroundings.

However, it was found that community houses were well-constructed and sturdy in spite of residents’ weak economic status, indicating that housing needs were given primacy over the need for food. The three basic individual needs, food, shelter, and clothing, must be met to achieve a satisfactory standard of living. Nevertheless, individuals sometimes encounter occasions when they have to decide on prioritizing one or two of the three. That is, some may prioritize food over shelter or clothing, while others may place greater importance on shelter. Some of the respondents described prioritizing household expenditures over nutrition. For example, one respondent explained: “We save for house repairs first, food for the children follows what we eat daily.” However, some are determined to balance all three needs proportionately. In this case, it is essential to guide communities to place greater focus on meeting children’s nutritional needs over other considerations.

In terms of water sources, 22.75% of families with stunted children depended on wells, rivers, or rainwater. Taking water from the last two sources is problematic, considering that water for drinking should ideally be groundwater from a specific depth, at an optimal distance from septic tanks, and that it should fulfill established chemical, physical, and microbiological standards. Novianti et al. [27] indicate that insufficient drinking water availability, in terms of quality and quantity, accounts for one stunting risk factor. A study conducted in Ethiopia highlights that improved access to clean water, together with facilities and the availability of sanitation equipment, can reduce the prevalence of diarrhea and stunting among children [32]. In other words, water quality should comply with health standards, preventing illnesses in children, averting delays in their growth, and maintaining their health.

In addition to ensuring proper access to quality drinking water, the implementation of hygiene and sanitation practices among caregivers should also be enhanced. Several habits, for example, washing hands at least five times a day before and after meals, after using the bathroom, after handling soiled diapers, and when cleaning infants following bowel movements, need to be introduced and intensified [27], particularly in Sukodono, where such practices are rare. Stakeholders could improve the community’s knowledge and practices related to child-rearing through training programs, campaigns or adequate facility and infrastructure provision for the public benefit.

2. Behavioral and cultural determinants

Many observational studies indicate that a high level of education among caregivers protects against stunting and undernutrition [33]. The demographic data from the respondents in the study showed that the educational level of the respondents was relatively low and that mothers had higher educational attainment than fathers. Sragen has adequate basic education facilities (up to high schools). It provides limited options for tertiary education, driving those wishing to pursue higher education to seek institutions in other cities or regions. Attending college outside the regency incurs higher expenses, which may further contribute to the low interest among the Sragen community. This is concerning, since children cared for by individuals with higher education are more likely to receive adequate nutrition to support their growth. Moreover, Nguyen et al. [34] state that children living in urban areas experience better growth than those in rural settings, largely due to parental education levels. Higher education also enhances individual awareness of health issues, leading to greater attention to factors affecting family health. Educated caregivers typically possess a variety of strategies for ensuring children consume nutritious foods. Similar findings reported by other researchers [9,19,35]. Furthermore, Chen et al. [35] note that grandmothers play a more significant role in providing nutrition for children than mothers or other caregivers. Based on the Chi-square analysis, parental income had an insignificant effect on healthy food knowledge (χ² = 0.043, df = 1, p = 0.836).

In general, caregivers’ knowledge in families with stunted children in Sukodono was considered good (Table 2). Respondents were aware of exclusive breastfeeding, complementary foods, the significance of diverse foods for children, and various protein and nutrient sources. Nonetheless, the respondents answered three out of 17 questions incorrectly, namely the appropriate timing for introducing complementary foods, the consumption of sugar by children, and the provision of ready-to-eat foods (junk food) to children. Three questions regarding breastfeeding and complementary feeding were posed, with one receiving a low score, implying the uncertainty of respondents, specifically when answering questions about complementary feeding. Such uncertainty was unlikely to have arisen if they had effectively applied their knowledge to behavioral practices or habits. Feeding practices were determined by perceptions of breast milk adequacy. One caregiver noted: “I thought giving porridge early would calm the baby, but breast milk alone seemed not enough.” Suhartatik et al. [36] concur that an individual’s knowledge does not necessarily translate into behavioral change. Upon reviewing the knowledge assessment in Table 2, it was inferred that the community’s knowledge was generally satisfactory. However, further identification of behavioral practices and sustained efforts to translate knowledge into action remain necessary.

**Table 2:** Caregivers’ Levels of Knowledge.

Nr.	Statements in the Questionnaire Concerning Healthy Behaviors and Knowledge	% Right Answers
1	Exclusive breastfeeding is the provision of breast milk only without other supplementary foods during the first six months of a baby’s life.	100.00
2	Complementary food is ideally introduced to babies at the age of four months.	73.02
3	Complementary food to breast milk is given after the baby is six months old or before six months if the baby has a special condition.	92.06
4	Complementary food to breast milk should contain sufficient nutrients, such as iron and vitamins, and be processed from foods consumed by the family daily.	99.47
5	Introducing a variety of foods to babies helps meet nutritional needs and reduces the risk of allergies.	98.41
6	Food containing iron, for example, beef liver, is important to prevent anemia in babies.	99.47
7	Protein from meat, fish, and eggs helps muscle growth and child development.	100.00
8	The consumption of added sugar in children is allowed and not restricted because they need it for energy.	56.61
9	Milk and dairy products contain calcium and protein that are good for children’s bones and teeth.	100.00
10	Giving high-salt food to children can increase the risk of high blood pressure later in life.	96.83
11	Fibers in food serve as nourishment for good bacteria and reduce the risk of constipation in children.	96.83
12	Junk food should be given regularly to children because they like it.	68.78
13	Healthy food portions for children varies from one to another and must be adjusted to their age and physical activities.	96.83
14	Sweet drinks, including soda and sweet fruit juice, can cause tooth decay and obesity in children.	92.59
15	Introducing children to seafood, including fish and shrimp, is important because it contains omega-3 fatty acids.	98.94
16	Healthy snacks, e.g., fresh fruit and nuts, are better than snacks high in sugar and fat.	99.47
17	Eating leisurely and not in a hurry (no more than 30 minutes for each meal) is important for good digestion.	100.00

In transforming knowledge into expected behaviors and ultimately into desired habits, the individuals concerned should understand the benefits of the desired behavioral changes. For example, in the case of promoting exclusive breastfeeding, education related to its long-term health benefits for infants and mothers and its implications for the future is imperative. In addition, strong motivation, a supportive environment from family and friends, and easy access to breastfeeding opportunities at any time and place can significantly support this initiative. Questions regarding whether mothers should wake their infants at feeding time should also be addressed. Prospective mothers need to have a comprehensive understanding of the breastfeeding process from A to Z.

Changes can commence with small steps, including educating caregivers on correct breastfeeding techniques, preparing mothers and infants for breastfeeding, and informing mothers how often breast milk should be provided. These small changes can then evolve into habitual practices [37]. The transition from minor attitude adjustments to established habits typically takes 18–254 days, 66 days on average, depending on supportive and inhibitory factors [38]. The transition from knowledge to attitude change, and eventually to habit, can be measured using the theory of planned behavior [39].

According to Tsegaye et al. [40], supportive factors such as spousal support, significantly influence changes in breastfeeding behaviors and the nutritional status of mothers. This illustrates that mere education is insufficient and that comprehensive support from the surrounding environment, policies regarding lactation spaces, minimization of inhibitory factors, and other measures are also essential. Local government support concerning resources and infrastructure, the provision of designated breastfeeding times for working mothers, and childcare facilities near workplaces further facilitate breastfeeding practices among mothers.

It is believed that educating caregivers can greatly influence behavioral changes in children [9]. Caregivers should hence be educated on foods considered appropriate and inappropriate for children under five years of age. The focal points of education should also be set on nutritional adequacy and the urgency of physical activities in promoting children’s motor development. The age of five is a crucial period for children’s growth and development; accordingly, their nutritional needs should be satisfied, and appropriate stimulations should be given to ensure optimal growth and development.

A healthy lifestyle plays a crucial role in determining an individual’s health status, encompassing various factors, such as dietary choices (eating patterns), nutritional adequacy, daily activities, physical activities, socioeconomic factors, psychological factors, and the influences of culture and the social environment [41–43]. A balanced diet that emphasizes the consumption of fruit and vegetables, whole grains, and adequate protein for individual needs also supports this. Likewise, being engaged in regular physical exercise will lead to a better nutritional status. Parents’ lifestyles greatly affect their children, in the same way that peers and individuals in the same environment influence each other.

In addition to lifestyle factors, socioeconomic conditions also affect one’s overall health. Individuals with a good economic standing have better access to healthcare facilities and a broader range of healthy food options, while families with lower incomes often face poor access to nutritious foods and healthcare services. Psychological factors, such as stress levels, mental health and sleep patterns, can also influence eating behaviors, thereby indirectly affecting nutritional status. For instance, stress may lead to overeating or, conversely, inadequate food intake. Under normal circumstances, the aforementioned factors affect an individual’s health at an acceptable level, but are likely to influence pregnant or breastfeeding women more significantly. Therefore, it is essential to pay particular attention to the lifestyles of such women. Beyond the factors previously discussed, cultural and social factors also have significant influences on the nutritional status of pregnant and breastfeeding mothers and their child-rearing practices.

Interviews revealed that caregivers often possessed accurate knowledge but faced cultural and social pressures that shaped their practices. For instance, one caregiver correctly defined exclusive breastfeeding as “only breast milk for six months,” yet reported introducing porridge at four months because elders in the household believed the child would “grow stronger with solid food.” Another caregiver acknowledged that junk food was unhealthy but allowed her child to consume packaged snacks, explaining that “the child cries if not given, and neighbors say it is normal.” These examples illustrate the gap between knowledge and practice, influenced by local beliefs, family expectations, and social norms, and underscore the need for interventions that address behavioral and cultural barriers alongside education.

As shown in Table 3, the Sukodono community has a high awareness of nutritional needs and hygiene. Respondents consider the importance of eating fruit, vegetables, whole grains, and iron, as well as practicing hand washing and maintaining oral hygiene. A hundred percent of the respondents agreed that exclusive breastfeeding could effectively enhance children’s nutritional status. Some acknowledged that physical activities and socializing outside the house were essential, while 79.89% contended that children should take part in more activities at home than outside. This discrepancy suggests a need for further education on the significance of physical activities for children. From a dietary perspective, respondents or families recognized that excessive consumption of added sugars and salt was detrimental to children’s health. Furthermore, they also perceived the importance of socialization and interaction within the family. The Chi-square analysis indicated that educational levels had a significant impact on lifestyle (χ² = 4.566, df = 1, p = 0.033). This suggests that caregivers with higher education are more likely to adopt health-promoting behavior, thereby influencing child nutrition and stunting outcomes.

**Table 3:** Identified Lifestyle of Families with Stunted Children.

No.	Lifestyle Parameter	% Respondents in Agreement
1	In addition to meat, fruit, vegetables, and grains are also good for children to consume.	98.41
2	Sufficient physical activity helps strengthen children’s muscles and bones.	98.94
3	Added sugar in canned juice and soda has an adverse effect on children’s health.	97.35
4	Exclusive breastfeeding for the first six months of life helps prevent stunting.	100.00
5	The risk of high blood pressure in children can be triggered by the consumption of high-salt food.	97.35
6	Teaching children the importance of washing their hands before eating helps prevent disease and stunting.	99.47
7	A diet rich in iron helps prevent anemia in children.	100.00
8	Children who actively play and socialize outside the house have a lower risk of stunting compared to children who stay at home continuously.	79.89
9	Consumption of grains can also maintain children’s digestion because of the fiber they contain.	100.00
10	The correct chewing process will help children’s digestion work better.	100.00
11	Children who are active in sports or art activities show better emotional control.	97.35
12	Introducing children to fermented foods, such as yogurt or tempeh, supports digestive health.	99.47
13	Avoiding exposure to cigarette smoke and air pollution helps maintain children’s lung health.	100.00
14	A child with full support and engaged in activities with the family tends to socialize with others better.	99.47
15	Children are taught that maintaining oral hygiene will prevent tooth and gum damage.	100.00
16	Children are taught that always wearing footwear when playing outdoors is a good habit.	96.83
17	Breakfast is the main thing that is good for children’s health.	99.47

Based on the data in Table 3, suggestions for the government include increasing the consumption of nutritious foods; promoting physical activities and social interaction; maintaining cleanliness and family health; and fostering commitment and support from the entire family. Relevant campaigns should be undertaken to encourage the consumption of fruit, vegetables, and whole grains in addition to meat, to ensure that children have a varied diet rich in iron. Furthermore, it is essential to note that one of the reasons for the high prevalence of stunting is the raised level of anemia among pregnant women. Based on the examination of the socioeconomic aspects, lifestyle, and knowledge of the Sukodono community, their knowledge levels were found to be satisfactory. Their social and economic conditions were also relatively good, although some aspects call for improvement.

As shown in Tables 2 and 3, most caregivers achieved scores in the “adequate” or “good” range (≥ 60% correct response), indicating relatively high levels of structured knowledge and reported lifestyle practices. Consistent with the research hypothesis, caregiver education levels were examined in relation to knowledge and feeding practices. Lifestyle practices, including sanitation and dietary habits, were analyzed and showed that although most households had access to latrines and clean water, gaps remained in daily hygiene and feeding behaviors. Maternal health indicators, such as age at childbirth and prenatal care, were also assessed, and demonstrated that younger maternal age at delivery and incomplete adherence to iron and folic acid supplementation were associated with a higher risk of stunting. These findings collectively address the research question by identifying socioeconomic, behavioral, and maternal health factors that contribute to stunting outcomes in Sukodono.

Nevertheless, the stunting rate in Sukodono remains high. Some aspects requiring further investigation or improvement include access to nutritious food, implementation of balanced diets, enhanced hygiene and sanitation practices within families, and a reduction in children’s exposure to pollutants, especially tobacco smoke. Campaigns highlighting the importance of education for families should also be pursued. Improving access to further education is vital because knowledge has been proven to be insufficient. Finally, relevant efforts should be made to ensure that such knowledge leads to behavioral changes and eventually becomes part of the community’s daily life.

3. Implications for policy and programming

The persistence of high stunting rates in Sukodono, despite adequate caregiver knowledge and national program coverage, underscores the need for locally adapted interventions. National supplementary feeding programs could be strengthened by integrating behavior-change communication, addressing household economic trade-offs, and engaging community leaders to challenge cultural norms around feeding practices. These adaptations would help bridge the gap between knowledge and practice, ensuring that national strategies are more effective in local contexts. By integrating these behavioral, cultural, and economic considerations, national programs can be more effectively tailored to local realities, thereby bridging the gap between knowledge and practice in everyday caregiving.

The apparent inefficiency of the Supplementary Feeding Program in Sukodono underscores that food supplementation alone cannot adequately address stunting. Our findings suggest that program effectiveness would be strengthened if supplementary feeding were combined with behavior change communication to bridge the gap between knowledge and practice, community-level education on household financial priorities to ensure resources are directed toward child nutrition, and improvements in water and sanitation to reduce infection-related growth faltering. By integrating these components, the program could more effectively respond to local contextual factors and provide actionable guidance for policymakers and program planners seeking to reduce stunting in Sukodono.

4. Limitations

This study has several limitations that should be acknowledged. First, the cross-sectional design does not allow for causal inference, and the associations observed should be interpreted as descriptive rather than predictive. Second, reliance on self-reported behaviors and recall may introduce reporting bias, particularly in relation to feeding practices and lifestyle indicators. Third, the study was conducted in a single sub-district (Sukodono), which may limit the generalizability of findings to other settings with different socioeconomic or cultural contexts. Nevertheless, these limitations are common in community-based field studies and do not undermine the descriptive value of the work, which provides important insights into local determinants of stunting and their implications for program design.

## Conclusions

Stunting in Sragen appears to be influenced by a combination of factors, including caregiver education, lifestyle practices, maternal health conditions, sanitation infrastructure, and access to healthcare services. While education and lifestyle showed significant associations, other determinants such as premature birth, water quality and hygiene practices also contributed meaningfully to the high prevalence of stunting. Therefore, a multifaceted approach is essential to address the complex interplay between these factors. Awareness of the importance of formal education still needs to be improved, as does the community’s lifestyle and mindset concerning the nutritional needs of children. Additionally, the infrastructure for a clean water supply to meet the community’s needs should be developed. Knowledge about parenting styles, healthy nutrition, and the significance of maternal health was shown to be adequate. However, due to the high rates of stunting, ongoing efforts are needed to change community behaviors related to child-rearing and healthy living. Certain superstitions and cultural practices within the community should be gradually altered to increase awareness of the factors contributing to the high prevalence of stunting. Education was found to positively correlate with lifestyle, but had no impact on healthy food knowledge. Similarly, family income did not influence caregivers’ knowledge of healthy food.

## Supporting information

S1 Fig Percentage of Stunted Children in Sukodono and Comparison with Other Districts in Sragen (N = 761,998 children). (TIF)

S2 Fig Prevalence of Stunting in Regencies in Central Java (%) (N = 12,450 children). (TIF)

S3 Fig Parental Information (Data represent N = 189 respondents). (TIF)

S4 Fig Prenatal and Birth Medical Information (Data represent N = 189 respondents). (TIF)

## References

[1] Ssentongo P, Ssentongo AE, Ba DM, Ericson JE, Na M, Gao X, et al. Global, regional and national epidemiology and prevalence of child stunting, wasting and underweight in low- and middle-income countries, 2006-2018. Sci Rep. 2021;11(1):5204. doi: 10.1038/s41598-021-84302-w 33664313 PMC7933191

[2] Puspasari D, Trihono, Thaha RA, Junadi P, Kusnanto H, Sugihantono A, et al. Indonesian health survey 2023 in numbers [Internet]. Jakarta: Kemenkes BPKP; 2023. 964 p. Available from: https://www.badankebijakan.kemkes.go.id/ski-2023-dalam-angka/

[3] Sartika AN, Khoirunnisa M, Meiyetriani E, Ermayani E, Pramesthi IL, Nur Ananda AJ. Prenatal and postnatal determinants of stunting at age 0-11 months: A cross-sectional study in Indonesia. PLoS One. 2021;16(7):e0254662. doi: 10.1371/journal.pone.0254662 34260622 PMC8279365

[4] Mustofa A, Suhartatik N. Stunting Control in Bendo, Sukodono, Sragen, Central Java. Jakadimas. 2024;2(1):42–7. doi: 10.33061/jakadimas.v2i1.10755

[5] Yani DI, Rahayuwati L, Sari CWM, Komariah M, Fauziah SR. Family Household Characteristics and Stunting: An Update Scoping Review. Nutrients. 2023;15(1):233. doi: 10.3390/nu15010233 36615889 PMC9824547

[6] Yazew T. Risk Factors of Stunting and Wasting among Children Aged 6-59 Months in Household Food Insecurity of Jima Geneti District, Western Oromia, Ethiopia: An Observational Study. J Nutr Metab. 2022;2022:3981417. doi: 10.1155/2022/3981417 35070448 PMC8776470

[7] Gholampour T, Noroozi M, Zavoshy R, Mohammadpoorasl A, Ezzeddin N. Relationship Between Household Food Insecurity and Growth Disorders in Children Aged 3 to 6 in Qazvin City, Iran. Pediatr Gastroenterol Hepatol Nutr. 2020;23(5):447–56. doi: 10.5223/pghn.2020.23.5.447 32953640 PMC7481058

[8] Forh G, Apprey C, Frimpomaa Agyapong NA. Nutritional knowledge and practices of mothers/caregivers and its impact on the nutritional status of children 6-59 months in Sefwi Wiawso Municipality, Western-North Region, Ghana. Heliyon. 2022;8(12):e12330. doi: 10.1016/j.heliyon.2022.e12330 36590498 PMC9798164

[9] Morgan EH, Schoonees A, Sriram U, Faure M, Seguin-Fowler RA. Caregiver involvement in interventions for improving children’s dietary intake and physical activity behaviors. Cochrane Database Syst Rev. 2020;1(1):CD012547. doi: 10.1002/14651858.CD012547.pub2 31902132 PMC6956675

[10] Mills JP, Mills TA, Reicks M. Caregiver knowledge, attitudes and practices regarding vitamin A intake by Dominican children. Matern Child Nutr. 2007;3(1):58–68. doi: 10.1111/j.1740-8709.2007.00066.x 17238936 PMC6860752

[11] Putri YSE, Putra IGNE, Falahaini A, Wardani IY. Factors Associated with Caregiver Burden in Caregivers of Older Patients with Dementia in Indonesia. Int J Environ Res Public Health. 2022;19(19):12437. doi: 10.3390/ijerph191912437 36231732 PMC9566301

[12] Muhrodji P, Wicaksono HDA, Satiti S, Trisnantoro L, Setyopranoto I, Vidyanti AN. Roles and Problems of Stroke Caregivers: A Qualitative Study in Yogyakarta, Indonesia. F1000Res. 2021;10:380. doi: 10.12688/f1000research.52135.1 35186263 PMC8822138

[13] Martina D, Kustanti CY, Dewantari R, Sutandyo N, Putranto R, Shatri H, et al. Advance care planning for patients with cancer and family caregivers in Indonesia: a qualitative study. BMC Palliat Care. 2022;21(1):204. doi: 10.1186/s12904-022-01086-0 36414948 PMC9682799

[14] Sutrisna A, Vossenaar M, Poonawala A, Mallipu A, Izwardy D, Menon R, et al. Improved Information and Educational Messages on Outer Packaging of Micronutrient Powders Distributed in Indonesia Increase Caregiver Knowledge and Adherence to Recommended Use. Nutrients. 2018;10(6):747. doi: 10.3390/nu10060747 29890670 PMC6024872

[15] Setiawan A, Fitriyani P, Istifada R, Shorey S. Healthcare Providers and Caregivers’ Perspectives on the Quality of Child Health Services in Urban Indonesia: A Mixed-Methods Study. Int J Environ Res Public Health. 2021;18(15):8047. doi: 10.3390/ijerph18158047 34360337 PMC8345802

[16] Indonesia S. Sragen in numbers 2024. Sragen: Statistics Indonesia; 2024. Available from: https://sragenkab.bps.go.id/id/publication/2024/02/28/e01f958a1e2e72be248d229c/kabupaten-sragen-dalam-angka-2024.html

[17] Anugraheni TD, Izzah L, Hadi MS. Increasing the Students’ Speaking Ability through Role-Playing with Slovin’s Formula Sample Size. J Studi Guru Pembelajaran. 2023;6(3):262–72. doi: 10.30605/jsgp.6.3.2023.2825

[18] Syafrawati S, Lipoeto NI, Masrul M, Novianti N, Gusnedi G, Susilowati A, et al. Factors driving and inhibiting stunting reduction acceleration programs at district level: A qualitative study in West Sumatra. PLoS One. 2023;18(3):e0283739. doi: 10.1371/journal.pone.0283739 37000777 PMC10065257

[19] Beal T, Tumilowicz A, Sutrisna A, Izwardy D, Neufeld LM. A review of child stunting determinants in Indonesia. Matern Child Nutr. 2018;14(4):e12617. doi: 10.1111/mcn.12617 29770565 PMC6175423

[20] Laksono AD, Wulandari RD, Ibad M, Kusrini I. The effects of mother’s education on achieving exclusive breastfeeding in Indonesia. BMC Public Health. 2021;21(1):14. doi: 10.1186/s12889-020-10018-7 33402139 PMC7786474

[21] Beluska-Turkan K, Korczak R, Hartell B, Moskal K, Maukonen J, Alexander DE, et al. Nutritional Gaps and Supplementation in the First 1000 Days. Nutrients. 2019;11(12):2891. doi: 10.3390/nu11122891 31783636 PMC6949907

[22] Keats EC, Haider BA, Tam E, Bhutta ZA. Multiple-micronutrient supplementation for women during pregnancy. Cochrane Database Syst Rev. 2019;3(3):CD004905. doi: 10.1002/14651858.CD004905.pub6 30873598 PMC6418471

[23] Naninck EFG, Stijger PC, Brouwer-Brolsma EM. The Importance of Maternal Folate Status for Brain Development and Function of Offspring. Adv Nutr. 2019;10(3):502–19. doi: 10.1093/advances/nmy120 31093652 PMC6520042

[24] Wiradnyani LAA, Khusun H, Achadi EL, Ocviyanti D, Shankar AH. Role of family support and women’s knowledge on pregnancy-related risks in adherence to maternal iron-folic acid supplementation in Indonesia. Public Health Nutr. 2016;19(15):2818–28. doi: 10.1017/S1368980016001002 27181394 PMC10270846

[25] Haque MA, Zaman Wahid B, Farzana FD, Tanvir Ahmed SM, Ali M, Naz F, et al. Influence of the Suchana intervention on exclusive breastfeeding and stunting among children aged under 6 months in the Sylhet region of Bangladesh. Matern Child Nutr. 2023;19(4):e13535. doi: 10.1111/mcn.13535 37244871 PMC10483947

[26] Kuchenbecker J, Reinbott A, Mtimuni B, Krawinkel MB, Jordan I. Nutrition education improves dietary diversity of children 6-23 months at community-level: Results from a cluster randomized controlled trial in Malawi. PLoS One. 2017;12(4):e0175216. doi: 10.1371/journal.pone.0175216 28426678 PMC5398527

[27] Novianti S, Huriyati E, Padmawati RS. Safe Drinking Water, Sanitation and Mother’s Hygiene Practice as Stunting Risk Factors: A Case Control Study in a Rural Area of Ciawi Sub-district, Tasikmalaya District, West Java, Indonesia. Ethiop J Health Sci. 2023;33(6):935–44. doi: 10.4314/ejhs.v33i6.3 38784492 PMC11111280

[28] Setiawan AS, Budiarto A, Indriyanti R. Eating behavior of adolescent girls in countries with a high prevalence of stunting under five: a systematic review. Front Psychol. 2023;14:1228413. doi: 10.3389/fpsyg.2023.1228413 38090175 PMC10711052

[29] Liao H-E, Deng Y-M. The Role of Caregiver’s Feeding Pattern in the Association between Parents’ and Children’s Healthy Eating Behavior: Study in Taichung, Taiwan. Children (Basel). 2021;8(5):369. doi: 10.3390/children8050369 34066688 PMC8151811

[30] Olajide BR, van der Pligt P, McKay FH. Cultural food practices and sources of nutrition information among pregnant and postpartum migrant women from low- and middle-income countries residing in high income countries: A systematic review. PLoS One. 2024;19(5):e0303185. doi: 10.1371/journal.pone.0303185 38723007 PMC11081330

[31] Ramulondi M, de Wet H, Ntuli NR. Traditional food taboos and practices during pregnancy, postpartum recovery, and infant care of Zulu women in northern KwaZulu-Natal. J Ethnobiol Ethnomed. 2021;17(1):15. doi: 10.1186/s13002-021-00451-2 33743760 PMC7981893

[32] Girma M, Hussein A, Norris T, Genye T, Tessema M, Bossuyt A, et al. Progress in Water, Sanitation and Hygiene (WASH) coverage and potential contribution to the decline in diarrhea and stunting in Ethiopia. Matern Child Nutr. 2024;20(Suppl 5):e13280. doi: 10.1111/mcn.13280 34738323 PMC11258769

[33] Li H, Yuan S, Fang H, Huang G, Huang Q, Wang H, et al. Prevalence and associated factors for stunting, underweight and wasting among children under 6 years of age in rural Hunan Province, China: a community-based cross-sectional study. BMC Public Health. 2022;22(1):483. doi: 10.1186/s12889-022-12875-w 35277139 PMC8917668

[34] Nguyen HT, Eriksson B, Petzold M, Bondjers G, Tran TK, Nguyen LT, et al. Factors associated with physical growth of children during the first two years of life in rural and urban areas of Vietnam. BMC Pediatr. 2013;13(1). doi: 10.1186/1471-2431-13-149 PMC3849555 24066791

[35] Chen Y, Guo Y, Wu Y, Medina A, Zhou H, Darmstadt GL. Maternal empowerment, feeding knowledge, and infant nutrition: Evidence from rural China. J Glob Health. 2024;14:04094. doi: 10.7189/jogh.14.04094 38845456 PMC11157471

[36] Suhartatik N, Lathif YA, Wulandari YW, Mustofa A, Handayani S. Changes in the understanding and behavior of elementary school children due to nutrition education. In: AIP Conference Proceedings. 2023;2431(1). doi: 10.1063/5.0114278

[37] Gardner B, Rebar AL, Lally P. How does habit form? Guidelines for tracking real-world habit formation. Cogent Psychol. 2022;9(1). doi: 10.1080/23311908.2022.2041277

[38] Lally P, van Jaarsveld CHM, Potts HWW, Wardle J. How are habits formed: Modelling habit formation in the real world. Eur J Soc Psychol. 2009;40(6):998–1009. doi: 10.1002/ejsp.674

[39] Moghaddam FG, Salmani F, Chahkandak FH, Norozi E. Is the Theory of Planned Behavior a good model for predicting salt consumption behavior in pregnant women? A structural equation modeling approach. J Educ Health Promot. 2023;12:197. doi: 10.4103/jehp.jehp_983_22 37545990 PMC10402779

[40] Tsegaye D, Tamiru D, Belachew T. Theory-based nutrition education intervention through male involvement improves the dietary diversity practice and nutritional status of pregnant women in rural Illu Aba Bor Zone, Southwest Ethiopia: A quasi-experimental study. Matern Child Nutr. 2022;18(3):e13350. doi: 10.1111/mcn.13350 35315583 PMC9218320

[41] Gherasim A, Arhire LI, Niță O, Popa AD, Graur M, Mihalache L. The relationship between lifestyle components and dietary patterns. Proc Nutr Soc. 2020;79(3):311–23. doi: 10.1017/S0029665120006898 32234085 PMC7663317

[42] Papadopoulou SK, Mantzorou M, Voulgaridou G, Pavlidou E, Vadikolias K, Antasouras G, et al. Nutritional Status Is Associated with Health-Related Quality of Life, Physical Activity, and Sleep Quality: A Cross-Sectional Study in an Elderly Greek Population. Nutrients. 2023;15(2):443. doi: 10.3390/nu15020443 36678316 PMC9862893

[43] Varela LM. Dietary Influence on Nutritional Epidemiology, Public Health and Our Lifestyle. Nutrients. 2023;15(11):2555. doi: 10.3390/nu15112555 37299518 PMC10255532

