# Supplementary figures and images for: Socioeconomic priorities and stunting in rural indonesia: A mixed-method study
Source: PLoS One. 2026 Mar 3;21(3):e0317329. doi: 10.1371/journal.pone.0317329 (PMC12956084; doi:10.1371/journal.pone.0317329)

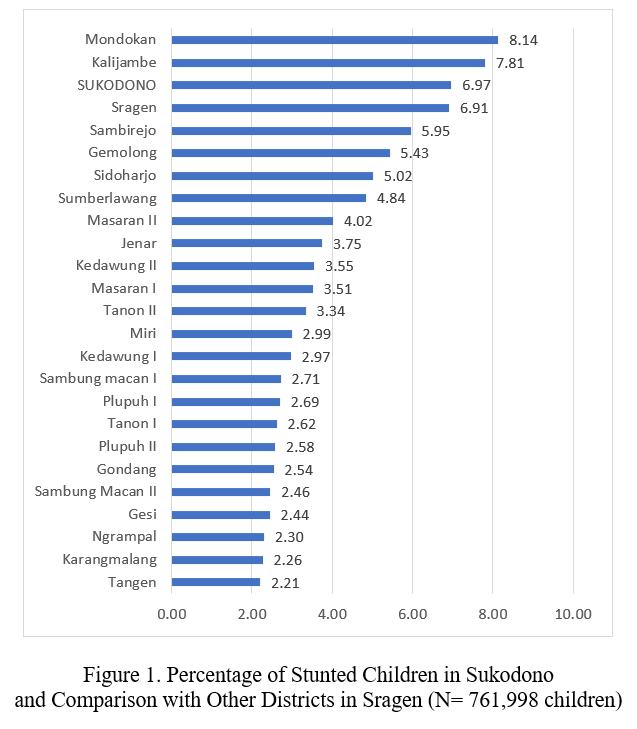

Supplement: S1 Fig — (TIF) [file pone.0317329.s001.tif]

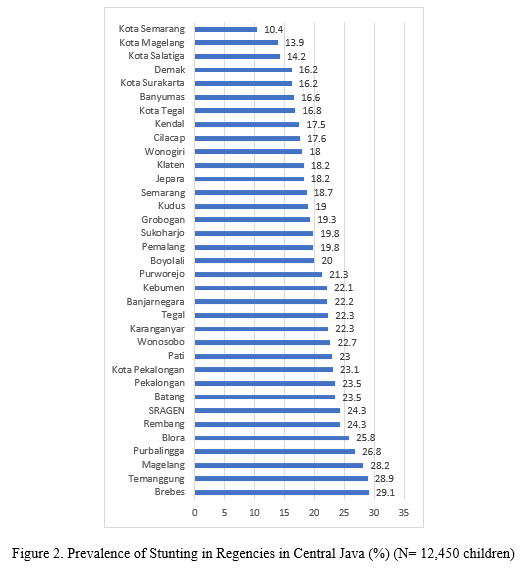

Supplement: S2 Fig — (TIF) [file pone.0317329.s002.tif]

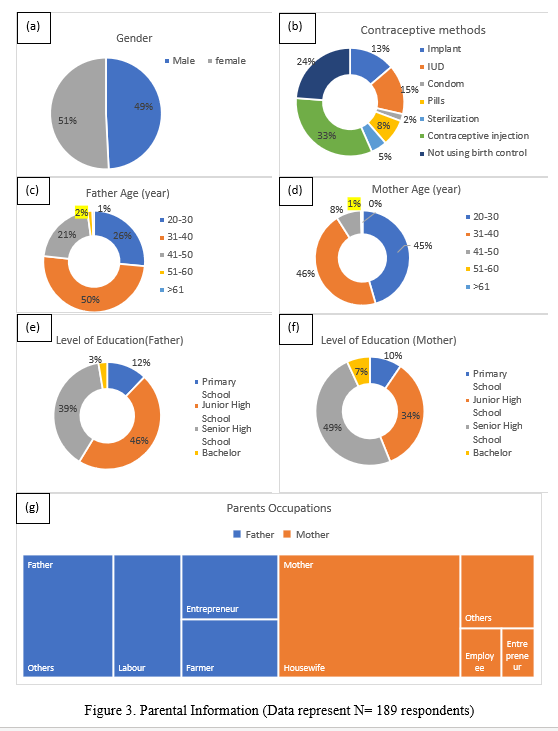

Supplement: S3 Fig — (TIF) [file pone.0317329.s003.tif]

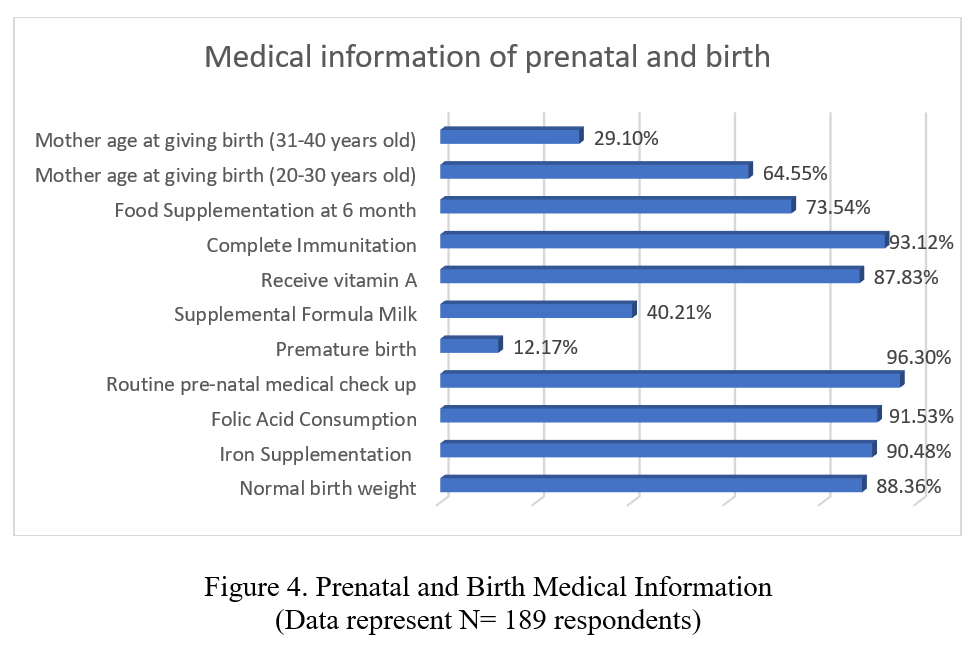

Supplement: S4 Fig — (TIF) [file pone.0317329.s004.tif]
